# Asclepias Tuberosa, Butter-Fly-Weed, Milk Weed, Pleurisy Root, White Root

**Published:** 1848-03

**Authors:** T. T. Lockwood

**Affiliations:** Buffalo, N. Y.


ART. III.—*Asclepias Tuberosa, Butter-fly-Weed, Milk Weed, Pleurisy Root, White Root.* By T. T. Lockwood, of Buffalo, N. Y.

The greatest care is necessary in collecting and preserving this root. As it is generally known in the country by the common name of Milk Weed, it is necessary to observe that this plant differs other species of Asclepias in not emiting a milky juice when wounded. The root should be collected about the first of October, cut in transverse slices, dried in the shade, and, as soon as sufficiently dried, pulverized and bottled. Pleurisy Root equalizes the circulation, produces copious expectoration and free diaphoresis, without inducing as much previous heat and excitement in the system as most other vegetable sudorifics. In the treatment of measles this root is often of essential service. When the rash is tardy in making its appearance, the cough harsh and dry, attended with pain in the eyes and fore part of the head, the warm decoction of this root may be given with marked good effect. It is decidedly the most valuable medicine I have ever administered to bring out the rash in all eruptive diseases, after the phlogistic state of the system has been properly attended to.

Owing to the intimate relation existing between the mucus membranes and the cutaneous exhalants, the Pleurisy Root is a most useful remedy in bronchitis, catarrh, and chronic diarrhœa of long standing. This root is especially serviceable in sub-acute and chronic rheumatic affections, when they are attended with a dry and harsh skin. In this complaint the warm decoction may be given alternately with the tincture of colchicum. Opium in any form has a tendency to produce congestion of the brain, and to lock up the secretions ; therefore, the Asclepias is preferable to Dover's Powder in all those low forms of fever in which there is a tendency to cerebral congestion, and where we wish to promote expectoration. In acute inflammation of the parenchyma, or of the serous membrane of the lungs, it will not do to rely upon the Pleurisy Root alone, but we should resort at once to active depletion. Asclepias Tuberosa possesses important medicinal properties. The warm decoction acts with as much certainty as a diaphoretic, as jalap does as a cathartic. It is peculiarly applicable to the diseases of children, as it posseses no disagreeable taste, or smell. I have frequently employed the Pleurisy Root in that continued and exhausting diarrhœa to which children are subject during the summer months, and generally with manifest advantage. In the latter complaint, the root should be boiled in fresh milk. Boil three drachms of this root in a quart of fresh milk down to one pint. Half an ounce of this is to be given every two or three hours. It generally excites a copious perspiration.

The White Root is applicable in every disease where diaphoresis and expectoration are to be promoted. The best mode of exhibiting this remedy is in the form of decoction. One ounce of the root may be boiled in three pints of water down to a quart, and given in doses of half a gill every half hour. Dose of the substance ten to fifteen grains.

FEBRUARY, 1848.

